# Under-Reporting of COVID-19 Cases Among Indigenous Peoples in Brazil: A New Expression of Old Inequalities

**DOI:** 10.3389/fpsyt.2021.638359

**Published:** 2021-04-12

**Authors:** Martha Fellows, Valéria Paye, Ane Alencar, Mário Nicácio, Isabel Castro, Maria Emília Coelho, Camila V. J. Silva, Matheus Bandeira, Reinaldo Lourival, Paulo Cesar Basta

**Affiliations:** ^1^Amazon Environmental Research Institute, Brasilia, Brazil; ^2^Coordination of the Indigenous Organizations of the Brazilian Amazon, Manaus, Brazil; ^3^University of Brasilia, Latin American Studies, Brasilia, Brazil; ^4^Lancaster Environment Centre, Lancaster, United Kingdom; ^5^Nature and Culture International, Brasilia, Brazil; ^6^International Institute of Education of Brazil, Brasilia, Brazil; ^7^National School of Public Health, Oswaldo Cruz Foundation, Rio de Janeiro, Brazil

**Keywords:** COVID-19, Indigenous Peoples, Brazilian Amazon, Indigenous health system, inequalities, under-reporting

## Abstract

**Objective:** To estimate the incidence, mortality and lethality rates of COVID-19 among Indigenous Peoples in the Brazilian Amazon. Additionally, to analyze how external threats can contribute to spread the disease in Indigenous Lands (IL).

**Methods:** The Brazilian Amazon is home to nearly half a million Indigenous persons, representing more than 170 ethnic groups. As a pioneer in heading Indigenous community-based surveillance (I-CBS) in Brazil, the Coordination of the Indigenous Organizations of the Brazilian Amazon (COIAB) started to monitor Indigenous COVID-19 cases in March of 2020. Brazil's Ministry of Health (MOH) was the main source of data regarding non-Indigenous cases and deaths; to contrast the government's tally, we used the information collected by I-CBS covering 25 Special Indigenous Sanitary Districts (DSEI) in the Brazilian Amazon. The incidence and mortality rates of COVID-19 were calculated using the total number of new cases and deaths accumulated between the 9th and 40th epidemiological weeks. We studied (a) the availability of health care facilities to attend to Indigenous Peoples; (b) illegal mines, land grabbing, and deforestation to perform a geospatial analysis to assess how external threats affect Indigenous incidence and mortality rates. We used the Generalized Linear Model (GLM) with Poisson regression to show the results.

**Results:** MOH registered 22,127 cases and 330 deaths, while COIAB's survey recorded 25,356 confirmed cases and 670 deaths, indicating an under-reporting of 14 and 103%, respectively. Likewise, the incidence and mortality rates were 136 and 110% higher among Indigenous when compared with the national average. In terms of mortality, the most critical DSEIs were *Alto Rio Solimões, Cuiabá, Xavante, Vilhena* and *Kaiapó do Pará*. The GLM model reveals a direct correlation between deforestation, land grabbing and mining, and the incidence of cases among the Indigenous.

**Conclusion:** Through this investigation it was possible to verify that not only the incidence and mortality rates due to COVID-19 among Indigenous Peoples are higher than those observed in the general population, but also that the data presented by the federal government are underreported. Additionally, it was evident that the presence of illegal economic activities increased the risk of spreading COVID-19 in ILs.

## Introduction

The advance of the novel coronavirus, which has already claimed more than one million lives globally, has hit the Indigenous populations of the Brazilian Amazon head-on. Indigenous Peoples bear a disproportionate brunt of the coronavirus pandemic as the result of the settler colonialization process that pushed them into a vulnerable situation (1). The history of Indigenous Peoples in Brazil, regardless of ethnicity, is marked by a series of epidemics caused by exogenous diseases, which have left a death trail in their wake, from the beginning of the colonization period until the current days (2). This process results from a deep and cruel history of subjugation and marginalization of Indigenous Peoples (3, 4), which they actively fight against since then. This is particularly troubling when considering the more than 100 free and autonomous Indigenous groups recently contacted or living in voluntary isolation (5), taking the difficulties to offer them appropriate and opportune health services into account.

The national Indigenous health system, coordinated by the Special Health Secretariat for Indigenous Peoples (SESAI) of the Brazilian Ministry of Health, has shown that it does not provide the necessary infrastructure to prevent and treat even ordinary diseases. Indigenous children and women present higher levels of malnutrition and anemia, among other morbidities (6), when compared to the Brazilian population as a whole (7, 8), as a direct effect of the inequalities expressed by health disparity (4, 9).

In addition to these chronic problems, the government's tally has been clearly under-reported. Based on the guidelines outlined in the National Policy for Indigenous Health Care, the federal government assures health assistance solely at the primary health care level and restricts the treatment to those living in Indigenous Lands (ILs) officially recognized by the Brazilian National Indigenous Foundation (FUNAI). Thus, the number of coronavirus cases does not include Indigenous Peoples living in cities as part of the tally of Indigenous Peoples infected, nor those who end up dying in their territories without receiving healthcare. Moreover, systemic racism issues persist, as their identity is sometimes denied, as some have been registered as *pardo*^1^ or brown instead of Indigenous.

In cases of coronavirus in which there are clinical complications, there is a need for a more complex care structure, which includes the use of medications that are not available in primary health care facilities, such as the provision of oxygen therapy through artificial respirators or hospitalization in Intensive Care Unit (ICU) beds. Therefore, Indigenous Peoples should be considered a high-risk group for COVID-19 and should receive the appropriate extra attention and catered care (11, 12). Unfortunately, the federal government is not fulfilling its role in effectively ensuring comprehensive health care for this population.

The current scenario among Indigenous Peoples in Brazil is severe and worrisome. The mortality rate registered among the Indigenous Peoples of the Amazon today is an indicative of a situation that can be catastrophic if an urgent and adequate strategy for treating these communities is not implemented within these regions. Therefore, the Indigenous organizations, particularly the Coordination of the Indigenous Organizations of the Brazilian Amazon (COIAB), set up an Emergency Action Plan that has several fronts, including the one responsible to monitor Indigenous cases of coronavirus in the Brazilian Amazon, that later would influence other Indigenous organizations in Brazil and the Amazon basin (13). In this sense, the Indigenous community-based surveillance (I-CBS) was established to keep track of Indigenous cases to contrast the government's tally, by combating the misclassification of Indigenous Peoples from data sets in order to have substantial data to orient health policies that attend their particular needs. Several Indigenous and indigenists organizations are dedicated to support the I-CBS work since March of 2020.

Taking into consideration the abovementioned factors that elucidate the context in which the federal government under-reporting takes place, in addition to existing health disparities and inequalities (9), this article aims to estimate the incidence and mortality rates as well as the lethality of COVID-19 among the Indigenous Peoples of the Brazilian Amazon. Additionally, we try to analyze how external threats that occur in traditional territories can contribute to the dissemination of the disease in Indigenous Lands, threatening lives and ancestral knowledge.

## Method

### The Indigenous Context in the Brazilian Amazon

The Brazilian Amazon is home to nearly half a million Indigenous population which, when translated into ethnical groups, represent one of the most socio-diverse regions in the world with more than 170 different nations (14, 15), in addition to those living in voluntary isolation, which represents the region with the highest number of Indigenous communities that chose to live freely and autonomously in the world (16). Each Indigenous nation has its own relation with its territories (17), which represents 98% of the total area of ILs in Brazil (18).

This great socio-cultural diversity is represented by COIAB and its robust organizational structure. Conceived in 1989, COIAB is ultimately the reference organization for the Indigenous Peoples of the Brazilian Amazon by representing its grassroots associations located in COIAB's sixty-four regions comprised in the states of the Legal Amazon. Moreover, COIAB's decision-making arrangement is supported by nine Indigenous state organizations, each representing their respective state in the region. Its long historical participation in debating and building public policies, which includes those of public health nature, paved the path that would conduct the efforts to combat the health disparities experienced by Indigenous groups (13).

As a pioneer in heading the I-CBS in Brazil, by the second half of March, COIAB started to monitor Indigenous coronavirus cases. This work is part of COIAB's Emergency Action Plan, aimed at preventing coronavirus from spreading in Indigenous communities and guiding public health policies to serve the areas and peoples that need more aid, in a collective effort to avoid losing more Indigenous lives. This document is an important tool of mobilization and planning to arrange the actions of COIAB and its partners. It was written with the following axis: communication, policy incidence, management of urgent actions of assistance and basic health care, food sovereignty, and Indigenous medicine (19).

Additionally, there was an urgent necessity to confront the information that was registered by governmental agencies, due to its colonialist vision of whom constitutes an Indigenous person. An administrative act issued by the Brazilian Ministry of Health defines that only those Indigenous persons living in their traditional territories have access to complete health care that respects their ethnocultural particularities (8). This norm creates a precedent by excluding Indigenous Peoples who live in urban areas from receiving treatment according to their cultural background.

**Figure 1:**
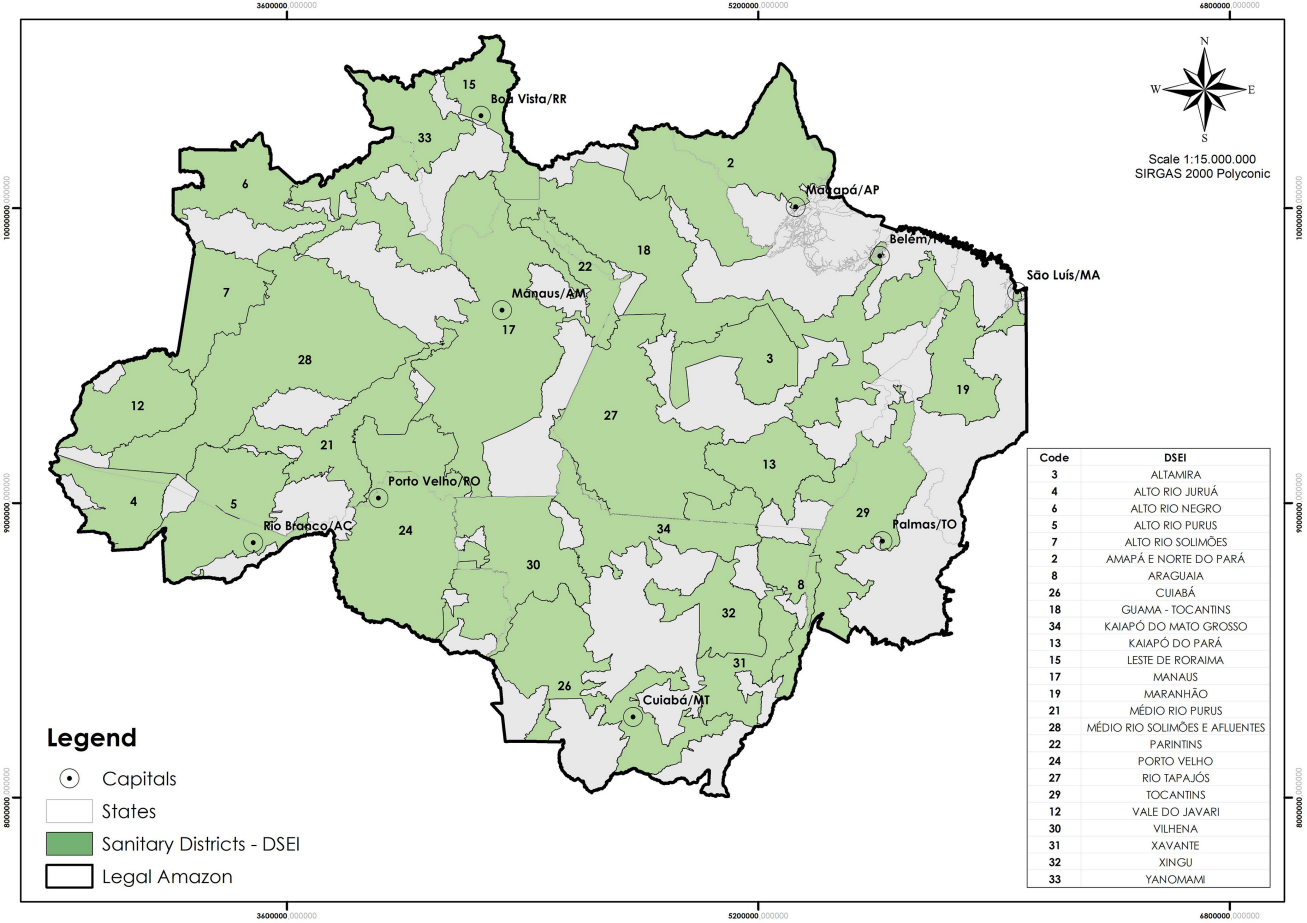
Coverage map of the twenty-five Special Indigenous Sanitary Districts (DSEIs) in the Amazon. Source: SESAI, Brazilian Ministry of Health.

In face of this challenge, COIAB and its network established a group dedicated to deal with coronavirus issues. Since then, they organize the information gathered by the twenty-five Special Indigenous Sanitary Districts (DSEI) located in the Legal Amazon region, comprehended within the nine states of the Amazon where COIAB acts directly through its member organizations. The DSEI are operational units whose range is defined not only by technical and geographical considerations, but also political relations, culture, and ancestral Indigenous population distribution (11). All ILs officially recognized by the government is served by one DSEI of reference; therefore, this is the area of analysis of this study (Figure 1).

### Sources of Data

#### COVID-19 Cases and Deaths

The Ministry of Health of Brazil was the main source of data regarding non-Indigenous confirmed cases and deaths, which we divided the analysis into the Brazilian population as a whole, and the nine states of the Legal Amazon^2^. We used the data released by SESAI regarding Indigenous cases; to compare with the tally compiled by the federal government, we used the information relentlessly collected by COIAB and their Indigenous leaders, Indigenous health professionals, and its partners in the Brazilian Amazon.

The health management of Indigenous Peoples in Brazil is under SESAI's responsibility. Its actions are developed through the Subsystem of Indigenous Healthcare (SASI), within the scope of the Brazilian Public Healthcare System (SUS), and are delivered by DSEI that are distributed over the entire national territory (11). As part of the Brazilian Ministry of Health, SESAI uses the same standard form to register every suspected case of coronavirus. Although this form asks for racial information, it does not include the option to inform about ethnicity or Indigenous territory of origin. Unlike the federal government, SESAI only releases total numbers of Indigenous coronavirus cases, without mentioning details about their age, gender, or origin. The daily-updated epidemiological bulletins are divided by DSEIs.

Such lack of detail makes it impossible to plan feasible and opportune actions to face coronavirus from spreading among Indigenous Peoples living in the Amazon. Thus, COIAB maintains a task force to qualify the information released by SESAI and to reinforce the necessity to amplify the spectrum of assistance by including the Indigenous living in urban areas. Together, they record all coronavirus cases amongst Indigenous Peoples not reported by the government, regardless of where the patient lives. Through this articulation, the I-CBS was constituted to fight the pandemic, given the importance of tracking the cases to control the spread of the disease. The reports were sent from the Indigenous and indigenists organizations to a focal point of COIAB, daily. Nonetheless, Indigenous leaders and local organizations sporadically informed COIAB directly about a confirmed case or fatality.

To guarantee there will not be double-counting, COIAB undergoes an internal check that compares the data in the bulletins issued by SESAI with the information passed on by the Indigenous leaders and organizations. Social networks were the main communication vehicle used by them to exchange information about cases and deaths. Additionally, COIAB keeps track of their partners' bulletins to complete the whole picture, as the case of Acre and Amapá states where indigenists organizations were fundamental to provide information. Furthermore, there is also an active Indigenous group that supervises the situation and validates its results.

COIAB's registry is presented by location - COIAB's region, state, DSEI, and municipality, when available -, the patient's situation (suspect, confirmed case, death), and ethnicity whenever possible. Later, COIAB elaborated a detailed bulletin to report the situation of the pandemic among Indigenous Peoples in the Amazon. In September, an app called *Alerta Ind*í*gena Covid-19* was launched to support the data collection work that had been done until then^3^.

Considering the ethical aspects of conducting research with human beings, the information used in this article comes from open sources when it comes to non-Indigenous Peoples. Regarding the Indigenous population data, we used the federal government's open sources and COIAB's registry, which is made public by their bulletins disclosed in their social media. COIAB was a proponent of this paper represented by two Indigenous leaders (VP, MN) and one indigenist (MC) as co-authors.

#### Environmental Variables

Aiming to analyze how external variables affect the Indigenous incidence, mortality, and lethality rates, we took into account territorial encroachment (20) and the availability of health care facilities to attend to indigenous peoples in their villages (11).

In terms of health care infrastructure, we considered the sum of Basic Healthcare Units (BHU) facilities administrated by SESAI^4^ per Indigenous population according to each DSEI. In addition, we measured the distance from the geographical center of the ILs to the nearest municipality with a hospital that has ICU beds, according to the Ministry of Health report^5^.

Additionally, we evaluated a set of three illegal activities detected within the ILs, by outlining (a) the existence of illegal mines, identified and mapped by the Amazon Geo-referenced Socio-environmental Information Network (RAISG)^6^, (b) the total area of illegally registered rural properties according to the Brazilian Rural Environmental Registry (CAR)^7^ as an indicator of land grabbing, and (c) the proportion of area deforested^8^ within the Indigenous territories and in a 10-kilometer buffer zone surrounding each IL, accumulated until 2019. The source for deforestation was the National Institute for Space Research (INPE) and the project PRODES, which monitor the clear-cutting of forests in the Legal Amazon area.

### Data Analysis

#### Descriptive Epidemiological Analysis of Incidence, Mortality, and Lethality Rate

The incidence rate of coronavirus was calculated using the total number of new cases accumulated between the 9th and 40th epidemiological weeks, as the numerator. In the same way, the mortality rate of coronavirus was calculated using the total number of deaths, accumulated during the aforementioned epidemiological weeks [9–40th (February 23 to October 03, 2020)], in the numerator. The denominator for incidence and mortality rates consisted of the total Brazilian population at risk during the calendar year 2019, multiplied by 100,000. Lastly, the lethality is expressed by percentage and contrasts the total number of deaths with the total confirmed cases.

For this study, the Brazilian population and the population of the Legal Amazon region is the one provided by the Court of Audit of the Union (TCU), in 2019. The Indigenous population is an estimate that used a geometric interpolation and extrapolation for the same year. The equation used to compute the growth of this population is the following:

$${\text{P}}_{\text{f}}={\text{P}}_{0}*{\left(1+\propto \right)}^{\wedge }\text{n}$$

*P*_*f*_ is the final population that takes into account the initial Indigenous population, *P*_0_, for each state of the Legal Amazon region, multiplied by *α* (*alfa*) as the Indigenous population growth rate from 2000 and 2010 (21), to the power of *n*, representing how many years from the initial population until 2019, to match with the estimated population for Brazil and the Legal Amazon.

#### Geospatial Analysis

The first step in the geospatial analysis was to use a correlation matrix to determine the connection between the environmental variables and the risk for an Indigenous person to get infected or die from COVID-19. Every variable was tested individually and those with a correlation higher than 0.2 were selected for further analysis (Table 1). The only exception for the correlation assessment was illegal mining, as it was represented as a binary variable.

**Table 1:** According to the correlation matrix, the incidence rate has a direct correlation with BHU, CAR, and illegal mining; the variables that showed a direct correlation with the mortality rate were BHU and deforestation accumulated.

	**Distance to** **UCI**	**Basic Healthcare Units (BHU)**	**Land grabbing (CAR)**	**Deforestation accumulated**
Incidence rate	−0.15	0.56	0.23	0.07
Mortality rate	−0.04	0.33	0.07	0.41

*Brazilian Ministry of Health, Amazon Network of Geo-referenced Socio-Environmental Information (RAISG); INPE; CAR/Brazilian Forest Service*.

We then used the GLM (Generalized Linear Model) with Poisson distribution to select the variables that together better predict incidence and mortality rates. In order to have comparable estimated coefficients, all input variables were converted to a 0 – 1 scale. The models were run from the most simple (null model) to the most complex (all variables added). The variables were added in turns to each model following a descending order based on the correlation coefficient. We assessed the models in pairs using the Analysis of Variance (ANOVA) to test if an addition of a variable improved the prediction of Indigenous cases and fatalities. Subsequently, to compare the models, we used the AIC index (Akaike information criterion) with the R package *rcompanion*. The AIC determines the best models prioritizing its goodness of fit (how well the model reproduces the data) and simplicity (the least number of variables). Finally, we selected the best models based on the least value of AICc, which are the following:

$$\begin{array}{l} \text{Incidence}\;\sim \;\text{BH}{\text{U}}_{\text{pop}}+\text{CAR}+\text{Mining},\;\text{family}="\text{poisson}"\\ \text{Mortality}\;\sim \;\text{Deforestation}+\text{BH}{\text{U}}_{\text{pop}},\;\text{family}=\text{"}\text{poisson}" \end{array}$$

*Incidence* and *Mortality* are the number of cases and deaths divided by population, multiplied by 100,000, respectively; *BHU_pop* is the amount of Basic Healthcare Units normalized by the population for each DSEI in the villages; *CAR* is the land grabbing area in hectares; *Mining* represents the illegal mining site area found within the DSEIs; *Deforestation* represents the proportion of the deforestation rates accumulated until 2019, within the ILs and a buffer zone of 10 kilometers around them. Different variables were selected for each model as correlations with incidence and mortality differed, and their combination is an outcome of the statistical selection which prioritizes the goodness of fit.

#### Vulnerability Index

The vulnerability index was designed for this study to analyze the variables chosen, given the aforementioned selected models, in two maps: (a) incidence index indicating the risk of contamination, combining access to health care services and exposure to the disease (represented by illegal mining and land grabbing) and (b) mortality index indicating the risk of death among the Indigenous population, which combines the access to healthcare infrastructure and deforestation variables. For each map, an index per DSEI was calculated based on the corresponding weight of each variable derived from the estimated coefficients in the GLM. The indices were obtained from the weighted sum of input variables, according to the following equations, and subsequently scaled from 0 to 1.

$$\begin{array}{l} \text{Index}(\text{a})=\sum \left(\left(\text{BHU}\text{∗}3,16\right)+\left(\text{Mining}\text{∗}1,20\right)+(\text{CAR}\text{∗}1,39)\right)\\ \text{Index}\;(\text{b})\;=\;\sum \left(\left(\text{BHU}\text{∗}2,05\right)+\left(\text{Deforestation}\text{∗}2,48\right)\right) \end{array}$$

## Results

### Incidence, Mortality and Lethality Rate

The pandemic caused by coronavirus stresses the differences within Brazilian society. Since the disease claimed its first victim in March, the total number of cases and deaths escalated. From then on, SESAI registered 22,127 cases, and COIAB's survey recorded 3,229 additional cases, totaling 25,356 confirmed cases, indicating a noticeable under-reporting of 14%, as a consequence of the official protocol that excludes Indigenous residents in cities, resumption areas, or territories affected by conflicts. The under-reporting regarding the number of deaths is much more alarming; SESAI reported 330, which represents less than half of all 670 deaths registered by COIAB up to October 1st, 2020. Regarding the incidence, mortality, and lethality rates, the proportion of the indigenous population affected by the novel virus is higher than the other groups in every regard (Table 2).

**Table 2:** Number of new confirmed cases and incidence rate of cases per 100,000 inhabitants; and the number of deaths and mortality rate per 100,000 inhabitants among the indigenous population of the Amazon (COIAB and SESAI), contrasting with cases and deaths in non-indigenous population by states of the Legal Amazon, and in Brazil on October 1st, 2020.

	**N^**°**^ of cases**	**N^**°**^ of deaths**	**Incidence rate per 100,000**	**Mortality rate per 100,000**	**Lethality rate**
COIAB	25,356	670	5,524	146.0	3.0%
SESAI	22,127	330	4,821	71.9	2.4%
Legal Amazon	941,425	22,379	3,247	77.2	1.5%
Brazil	4,906,833	145,987	2,335	69.5	2.6%

*Sources: COIAB, SESAI, and Brazilian Ministry of Health*.

**Figure 2:**
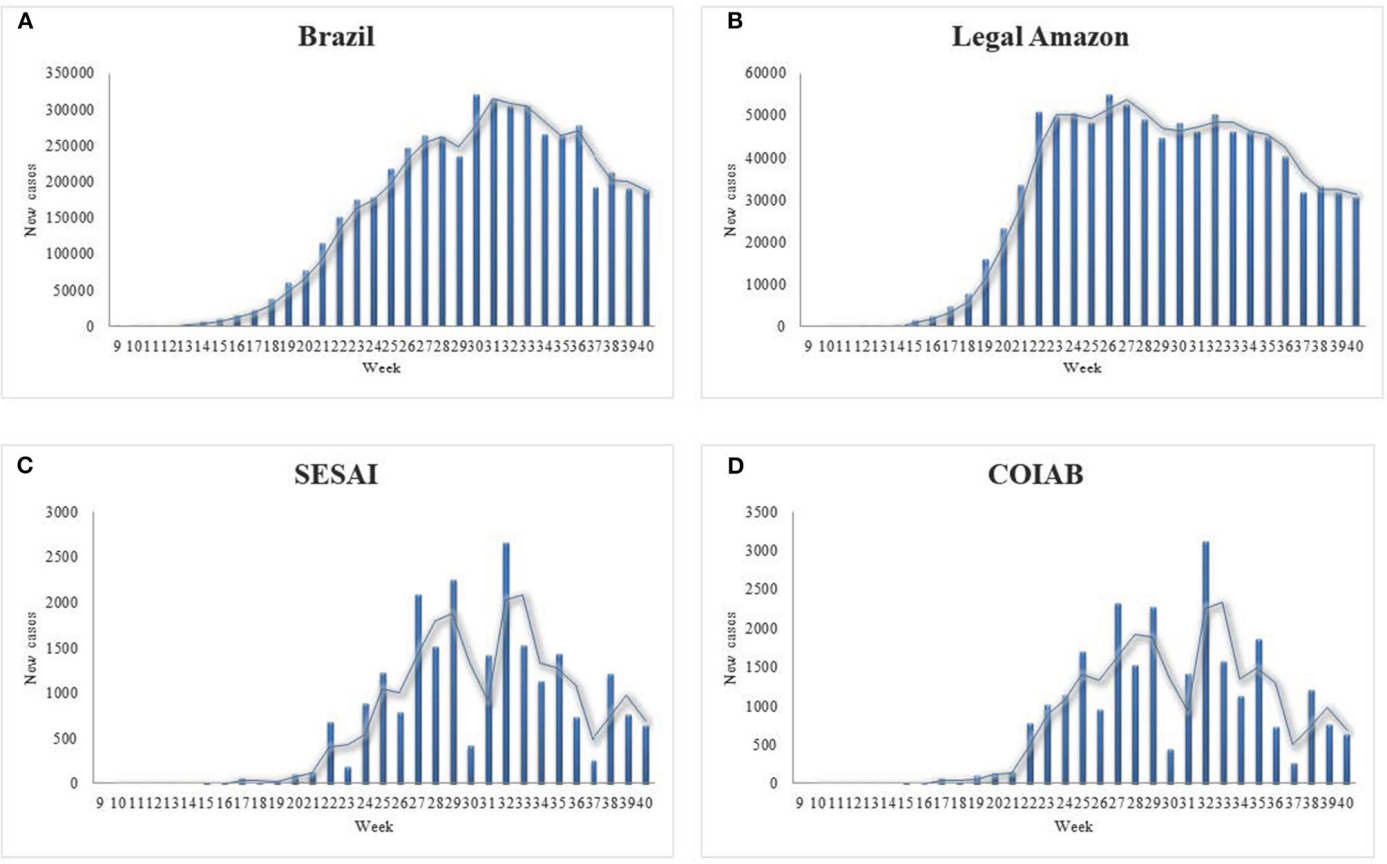
New confirmed cases from the 9th to the 40th epidemiological week of 2020, for **(A)** Brazil; **(B)** Legal Amazon; **(C)** for Indigenous Peoples cases according to COIAB; and, **(D)** Indigenous Peoples cases according to SESAI. Sources: COIAB, SESAI, and Brazilian Ministry of Health.

It should be noted beforehand that the total numbers presented in the graphs are significantly different, considering the new cases by epidemiological week (Figure 2). Nevertheless, the results indicate the distinct contamination dynamic for Indigenous Peoples when compared to the remaining groups. Concerning the total cases in Brazil, it is possible to outline the trajectory of the spread of the disease over time, which has led to almost five million cases by October 1st (Figure 2A). The Legal Amazon's tally follows a similar path to Brazil's, characterized by a sharper initial curve (Figure 2B). In contrast, the Indigenous new cases are distinguished by their particular fluctuation (Figures 2C,D). Although the Legal Amazon is the same region where the Indigenous population represented in this study lives, the trends shown differ drastically for both.

**Figure 3:**
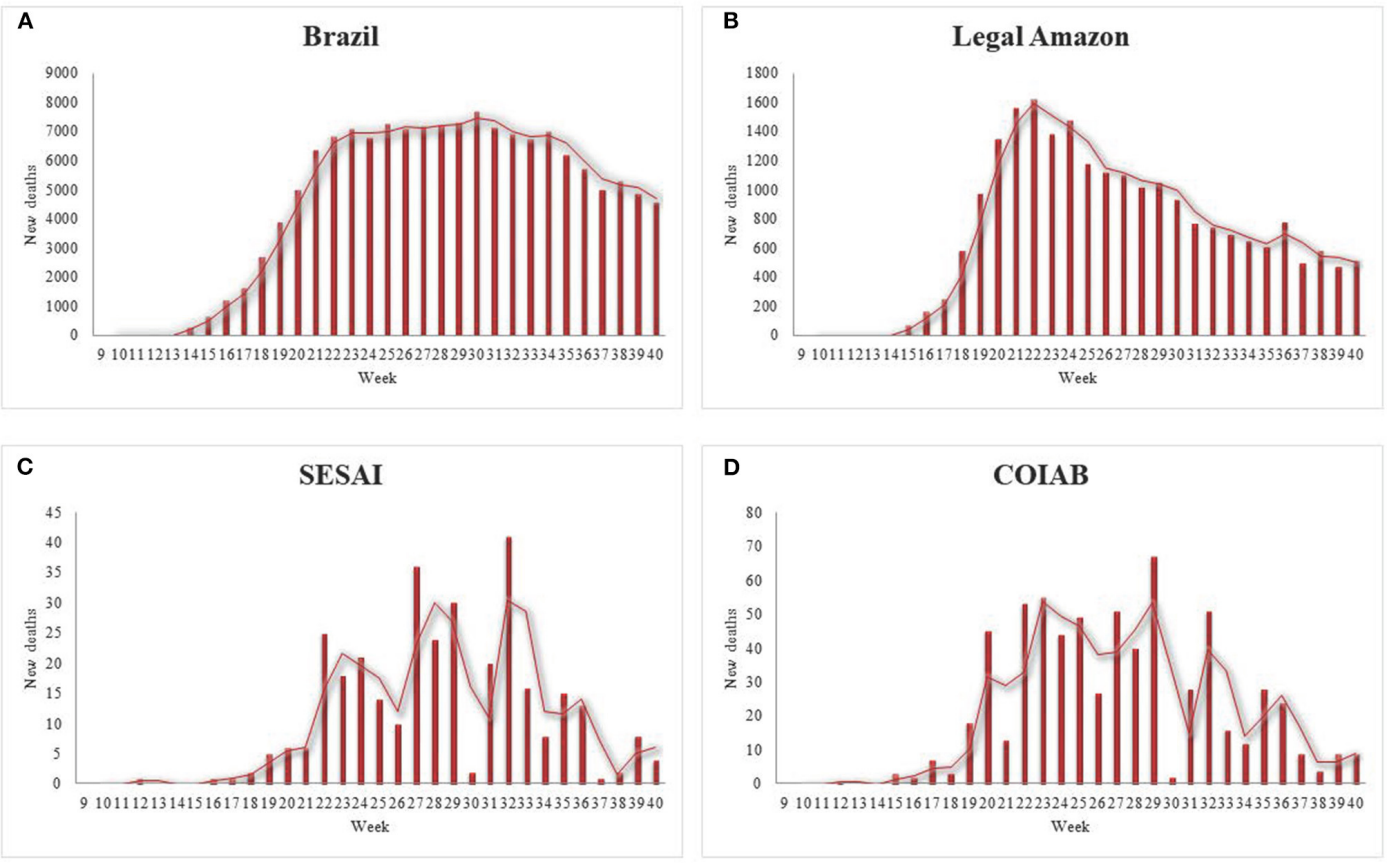
New deaths from the 9th to the 40th epidemiological week of 2020, for **(A)** Brazil; **(B)** Legal Amazon; **(C)** for Indigenous Peoples' deaths according to COIAB; and, **(D)** Indigenous Peoples' deaths according to SESAI. Sources: COIAB, SESAI, and Brazilian Ministry of Health.

Like the weekly report on new cases, the data presented for deaths showcase the specificities regarding Indigenous Peoples' condition (Figure 3). The cases in Brazil mirror its deaths (Figure 3A). The data demonstrate that after the curve peaked by the 22nd epidemiological week, the number of deaths per week remained stable, followed by a decrease in the latest weeks examined. The Legal Amazon presents similar features as those observed for Brazil in its entirety (Figure 3B). In contrast, the graphs for Indigenous deaths exhibit an oscillation in the number of fatalities per week, resembling the values for confirmed cases, as the virus spread throughout the communities (Figures 3C,D). It is noticeable that in the first weeks, COIAB's tally accounted for a larger amount of deaths caused by coronavirus in comparison with SESAI's reports, which indicates an expressive under-reporting.

**Figure 4:**
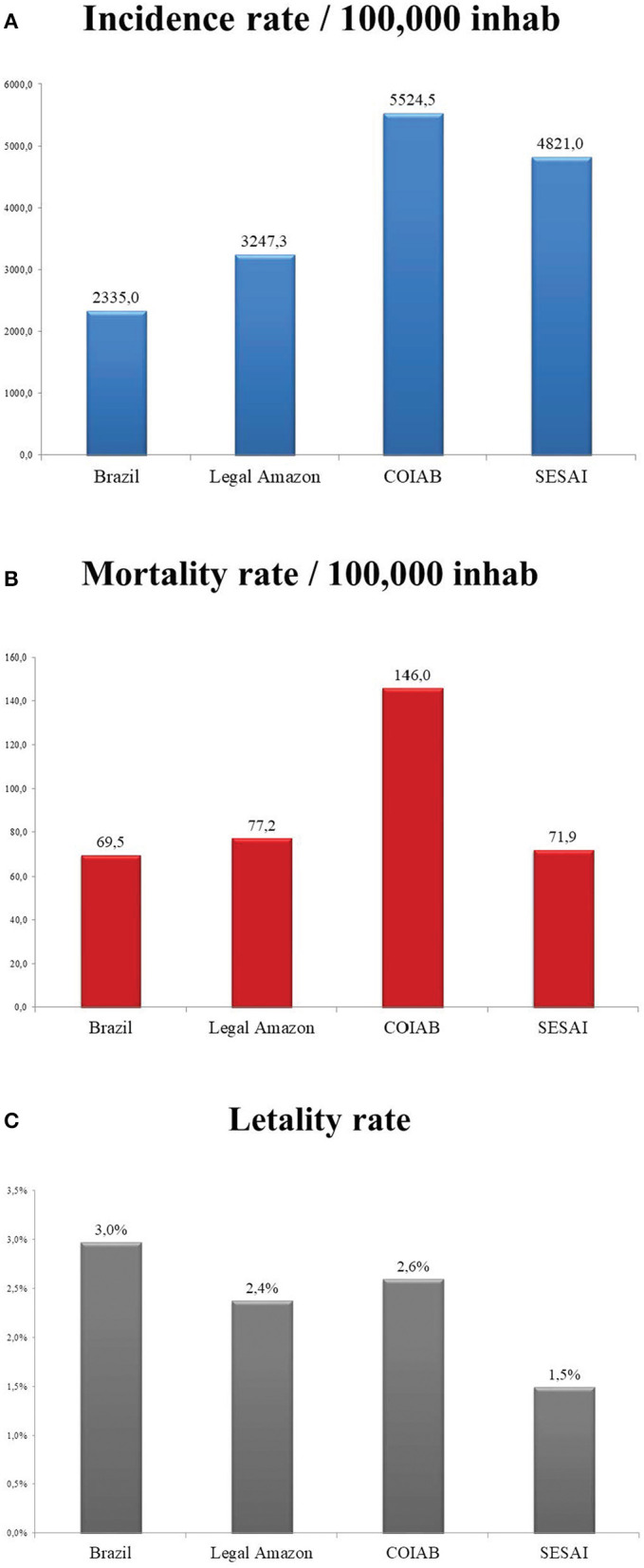
**(A)** Incidence rate per 100,000 inhabitants for Brazil, Legal Amazon, COIAB, and SESAI; **(B)** Mortality rate per 100,000 inhabitants for Brazil, Legal Amazon, COIAB, and SESAI; **(C)** Lethality for Brazil, Legal Amazon, COIAB, and SESAI, calculated for the 40th epidemiological week of 2020. Sources: COIAB, SESAI, and Brazilian Ministry of Health.

**Figure 5:**
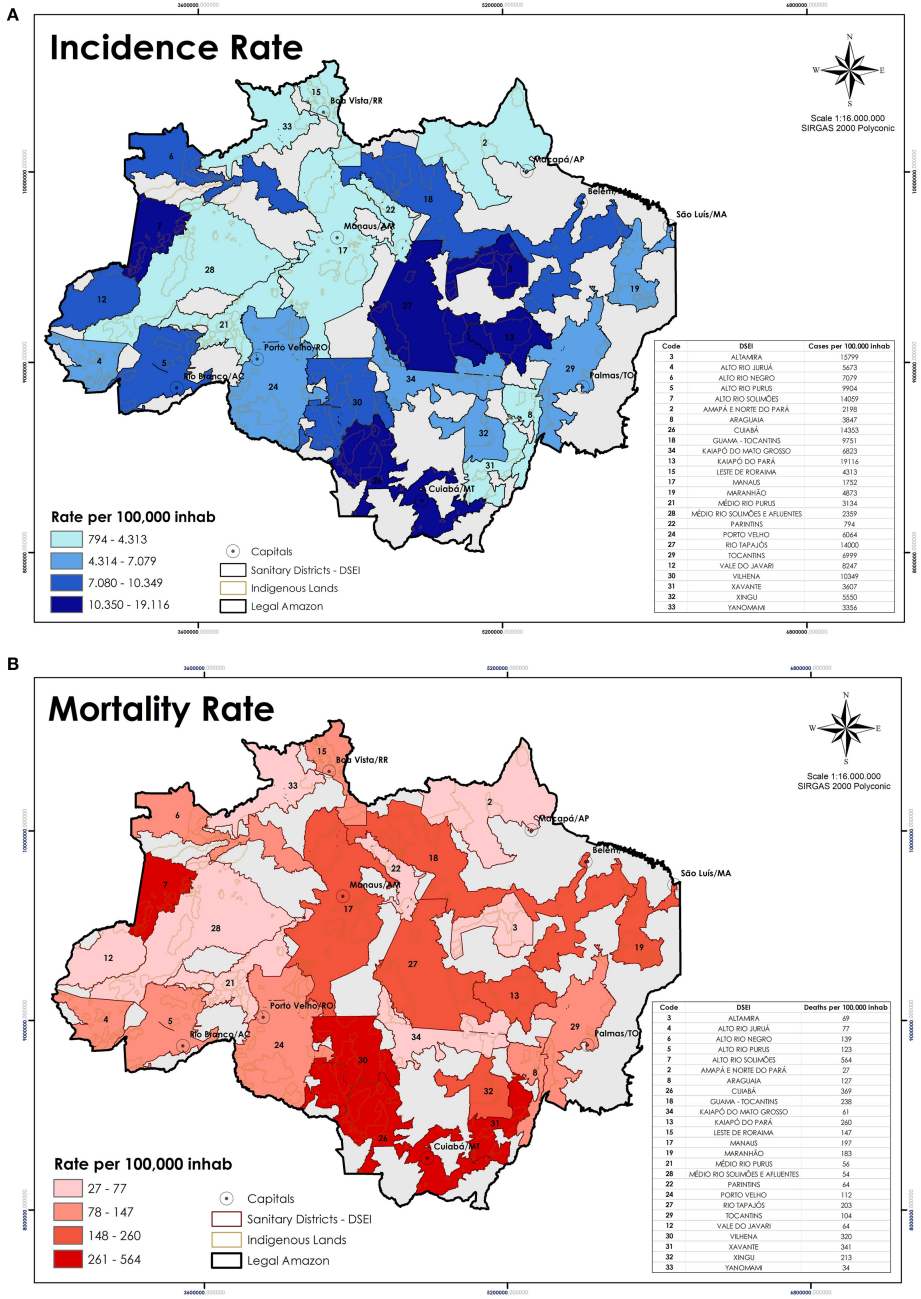
Each map shows the situation of the **(A)** Incidence rate; and **(B)** Mortality rate of the Indigenous population in the Amazon, distributed by DSEI, on October 1st. Source: COIAB.

According to COIAB's report, by the 40th epidemiological week, the incidence rate of COVID-19 per 100,000 inhabitants among the Indigenous population was 136% higher than Brazil's rate (Figures 4A, 5A). Likewise, this same metric was 70% larger for Indigenous Peoples than for the Legal Amazon region (Figure 4A). SESAI's data follows the same path; according to its database, the incidence rate for Indigenous communities is 106% higher than the Brazilian rate and 48% higher than the one observed in the Legal Amazon.

The Indigenous mortality rate per 100,000 inhabitants, according to COIAB's data, discloses a comparable situation to what was previously presented: it is 110% higher than the Brazilian average and exceeds the Legal Amazon mortality rate by 89% (Figures 4B, 5B). However, SESAI's record brings a different narrative that may reflect the under-reporting of deaths among Indigenous communities (Figures 3C,D). According to them, the mortality rate for Indigenous Peoples is lower than the Legal Amazon, and only 3.5% higher than Brazil's rate. The lethality also indicates a serious under-reporting as SESAI's values are 51% smaller than COIAB's numbers (Figure 4C).

The following maps enable a better understanding of the dispersion of coronavirus, previously presented in the graphs above (Figures 2C, 3C). Each area responded differently to the disease (Figure 5), with some severely impacted by the loss of several knowledge-bearers in their community, as the DSEI *Xavante* in the Southeast of the Mato Grosso state (Figure 5B), which reinforces the findings of previous studies (11). Another DSEI that showed a high level of incidence and mortality rates was the neighboring *Cuiabá* (Figure 5A). One DSEI sticks out from the trend noticed in its region, especially concerning the mortality rate, which is the *Alto Rio Solimões*. Located in the Northwest of the Amazonas state, this Sanitary District has twenty-seven ethnical groups, it has the fourth-highest incidence rate and it is first in mortality rate, which got to this level in the first period of contagion due to the contact of an infected health professional with the local communities (22).

### Vulnerability Index

**Figure 6:**
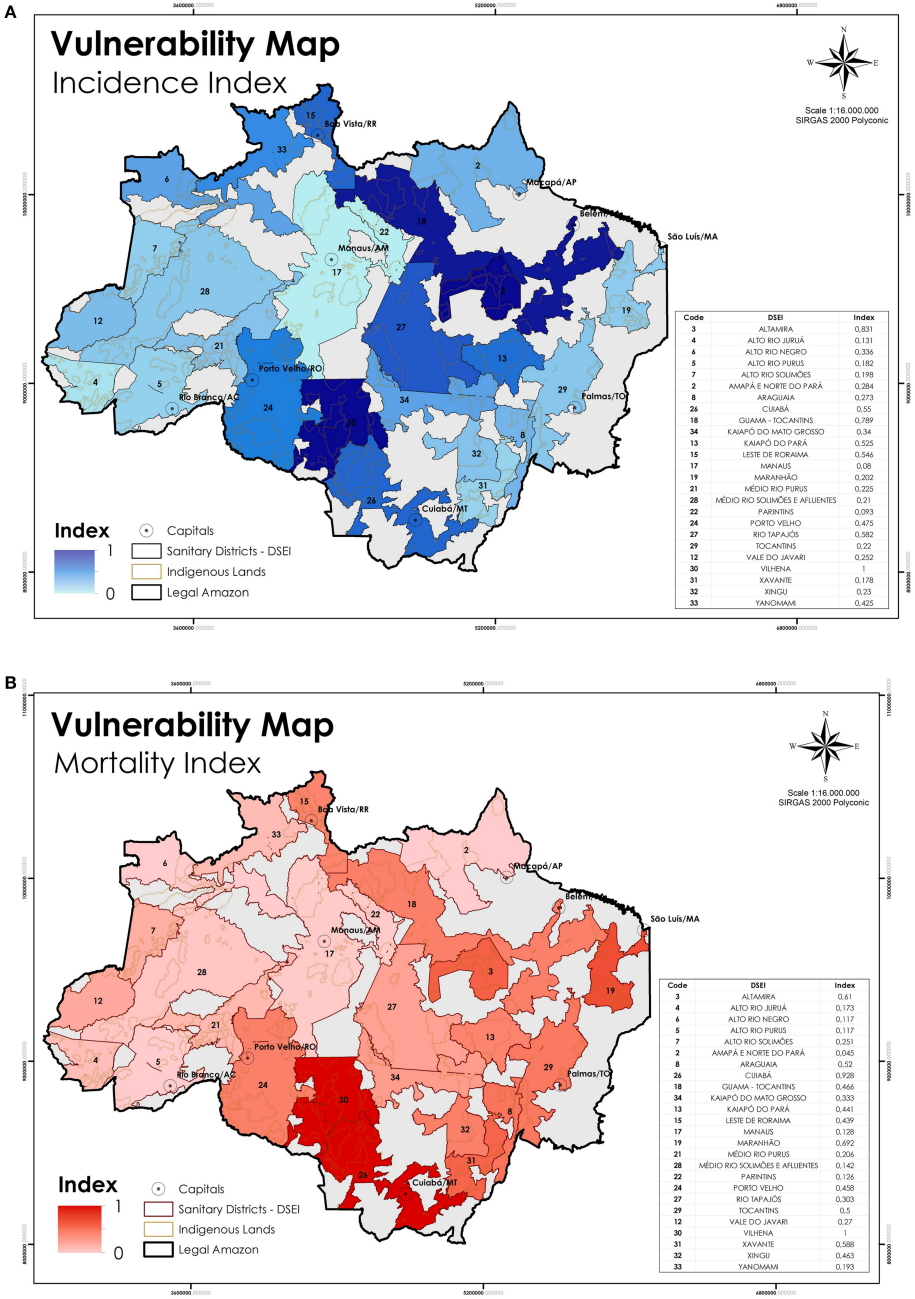
**(A)** DSEIs requiring greater attention to the risk of Indigenous populations getting infected or **(B)** die by COVID-19 due to external factors. Sources: Brazilian Ministry of Health, Amazon Network of Georeferenced Socio-Environmental Information (RAISG); INPE; CAR/Brazilian Forest Service.

According to the results obtained by the GLM tests, we calculated the weight of the sum of the variables by DSEI, by vulnerability to infection and death (Table 3). The aforementioned indices were the basis to structure the vulnerability maps (Figure 6), its indicator varies from 1 - those highly endangered - to 0 – those least at risk.

**Table 3:** Indices per DSEI, according to the sum of variables tested.

**Code**	**DSEI**	**Vulnerability index Incidence 0-1**	**Vulnerability index Mortality 0-1**
3	ALTAMIRA	0.831	0.61
4	ALTO RIO JURUÁ	0.131	0.173
6	ALTO RIO NEGRO	0.336	0.117
5	ALTO RIO PURUS	0.182	0.117
7	ALTO RIO SOLIMÕES	0.198	0.251
2	AMAPÁ E NORTE DO PARÁ	0.284	0.045
8	ARAGUAIA	0.273	0.52
26	CUIABÁ	0.455	0.928
18	GUAMA - TOCANTINS	0.789	0.466
34	KAIAPÓ DO MATO GROSSO	0.34	0.333
13	KAIAPÓ DO PARÁ	0.525	0.441
15	LESTE DE RORAIMA	0.546	0.439
17	MANAUS	0.08	0.128
19	MARANHÃO	0.202	0.692
21	MÉDIO RIO PURUS	0.225	0.206
28	MÉDIO RIO SOLIMÕES E AFLUENTES	0.21	0.142
22	PARINTINS	0.093	0.126
24	PORTO VELHO	0.475	0.458
27	RIO TAPAJÓS	0.582	0.303
29	TOCANTINS	0.22	0.5
12	VALE DO JAVARI	0.252	0.27
30	VILHENA	1	1
31	XAVANTE	0.178	0.588
32	XINGU	0.23	0.463
33	YANOMAMI	0.425	0.193

*Sources: Brazilian Ministry of Health, Amazon Network of Georeferenced Socio-Environmental Information (RAISG); INPE; CAR/Brazilian Forest Service*.

The maps indicate the areas where Indigenous Peoples are more exposed to the virus according to the outcomes (Figure 6A). The ILs potentially exposed to COVID-19, according to the variables examined are located at the DSEI *Vilhena, Altamira, Guamá-Tocantins, Rio Tapajós*, and *Cuiabá*. Concerning the greater risk for one infected person to die from coronavirus, the results show that the DSEIs *Vilhena, Cuiabá, Maranhão, Altamira*, and *Xavante* are expected to face a higher number of losses (Figure 6B). Each region requires crafted individualized attention, as they have their own socio-cultural, geographical, and demographical dynamic.

## Discussion

Inequalities in access to healthcare are a historical reality for Indigenous Peoples in many countries (23, 24), and Brazil is no exception (8). Mismanagement and disregards by WHO guidelines by the Federal authorities become evident on the actual results verified by COIAB's I-CBS on COVID-19 incidence and mortality rates among Indigenous Peoples compared to the government tally for Indigenous and the Brazilian population at large. The data presented by the federal government not only under-reports but also reinforces cultural assimilation attempts embedded in structural racism, by not accounting for indigenous people living in cities and denying their identities. However, the administration's handling of environmental stressors such as illegal economic activities (i.e., gold miners, loggers, land grabbers) on IL increased the risk of spreading coronavirus in all nine states of the Brazilian Legal Amazon. In one fell swoop, purposely threatening the lives and ancestral knowledge of different ethnic groups living in the Amazon region (25).

Our results indicate that Indigenous Peoples in the Legal Amazon are 136% more affected by COVID-19 than the rest of the country. Whilst mortality rate is 110% higher among Indigenous Peoples than the general population. Significant discrepancies between Legal Amazon states morbidity rates among Indigenous Peoples obtained by our study, correlated to settler colonization policy causing environmental stressors. Therefore, the spread of the coronavirus deserves maximum attention from health agencies, as confirmed by this study and COIAB's Emergency Action Plan. However, this urgency was not translated into combating the external sources of infection (26). It is key to halt contact with outsiders to stop the contamination cycle (20).

External agents carry the virus into the Indigenous communities at a faster pace than the government's ability to respond to the disease (27).

The GLM tests showed a direct correlation between the occurrence of illegal activities within the ILs and a high incidence rate, notably illegal mining and land grabbing. The current context is, therefore, grave. In addition to the external factors that threaten the health of Indigenous Peoples, there is still a lack of swift and widespread unequal medical care system. Reflected in our results by the amount of Basic Healthcare Units per population and the direct correlation with the incidence and mortality rates. A clear sign that the COVID-19 pandemic worsens an already harsh situation that Indigenous Peoples face.

However, it is essential to recognize some aspects of the current Indigenous health system. Conceived collaboratively by the Brazilian Indigenous movement, SESAI has been widely acknowledged as a major achievement and a step forward toward a health system that provides culturally safe and responsive services better able to address health inequities. This endeavor has played a major role in guaranteeing Indigenous participation in decision-making processes regarding health issues (28), and it has achieved great progress toward the implementation of the Brazilian public healthcare system (29). The data gathered by I-CBS indigenous network urged the administration to allocate to Indigenous Peoples, priority access to vaccines, through DSEIs and the National immunization program.

Managed by the federal government, this system has yet to improve. As the pandemic demonstrates, there are several gaps to be filled. An exemplary model is how the under-reporting gap that hides a bigger issue and hinders the combat of the pandemic was flagged by COIAB's I-CBS and is confronting SESAI's database regarding COVID-19 information. These shortages could be avoided by integrating civil society organizations as decision-making partners, in the opposed direction of the administration's policy.

Some enshrined rights have a sluggish pace toward transforming public policies into effective and efficient affirmative actions, while social inequalities prevent Indigenous Peoples from seeking medical care (8). For instance, many who live in their communities have to travel long distances to the nearest hospital for treatment. The average distance from one IL to a city with ICU beds is 271 km, which can reach up to more than 700 km, as the case of the DSEI *Alto Rio Negro* (30).

More than half of the Indigenous population in Brazil live in the Amazon (21). Of those, it is estimated that around 95,000 are living in urban areas, none of them covered by SESAI. Moreover, these people are not counted in the coronavirus government statistics as Indigenous, despite their constitutional entitlement, as well as it is not possible to follow their health conditions. A legal resolution in force limits SESAI's assistance to those Indigenous persons living in ILs officially demarcated by the federal government (8).

Indigenous ethnicity is also denied when the registration identifies them as *pardos*, a “whitening” leftover of Brazil's colonial (ist) past (10, 31). This severe situation is masked by the information reported by federal agencies: SESAI's lack of registered cases and deaths of Indigenous Peoples indicates a lethality rate 57% lower than COIAB's data. Such mismatch between registration of confirmed cases and deaths hinders the enactment of public policies to prevent further contamination and deaths, increasing a mortality trend already high among Indigenous Peoples (14).

During the pandemic, the Indigenous movement achieved a remarkable legal milestone. One was Law No. 14021, a collective process led by Indigenous and indigenists organizations, and political parties, that had the lawyer Joênia Wapichana, the first Indigenous woman elected as a federal deputy in Brazil, as the head of the process^9^. In the same direction, the jurisdictional act promoted by the Brazilian Indigenous Peoples Articulation (APIB) and six political parties, addressed to the Brazilian Supreme Court (STF)^10^ urging the implementation of a national plan to combat and monitor the pandemic among Indigenous Peoples. The first was approved to establish emergency actions to combat the advance of COVID-19 in Indigenous communities^11^, while the second demanded from the federal government the Indigenous right to exist. Although these were historical acts, the main loophole for Indigenous communities is still unresolved (32). Once again, the structural racism long experienced by Indigenous Peoples rears its ugly head (31, 33, 34).

The variables assessed in this study are indicative of the main threats to Indigenous lives. Previous works have pointed out the dangers of opening Indigenous territories to foreign actors (11, 20, 25, 27). Nevertheless, the vulnerability index is empirical and has limitations. The case of the DSEI *Alto Rio Solimões*, for instance, stands out, as the virus entered the community via a health professional who was infected, leading to an outbreak in the following weeks (22).

## Conclusions

Unfortunately, history repeats itself. Indigenous Peoples have endured violence throughout their past and until the present day. Their exposure to several diseases and conflicts has devastated many ethnic groups (35, 36), and COVID-19 appears to be a perfect storm in this regard (20, 27).

In this study, we conducted an analysis of public data on the situation of COVID-19 throughout Brazil until the 40th epidemiological week, closed on October 3rd, 2020. At that time, there was an expectation that the pandemic would be controlled, as the graphs pointed to a slight downward trend, considering the moving average of new cases and deaths.

However, a few weeks later, after the municipal elections in mid-November, both the number of cases and the number of deaths rose again. Today (middle-February, 2021), we can say that we are experiencing the peak of the second wave of the pandemic in Brazil, especially in the state of Amazonas. The encouraging news is that the available clinical trials (37) report that vaccines in phase three testing from international consortia are safe and effective.

Nevertheless, the federal government under the administration of Jair Messias Bolsonaro, who holds the sad title of the worst world leader in the management of the COVID-19 pandemic (38–40), has not prepared to offer vaccines to the population. Under Bolsonaro's guidance, Brazil not only did not join the consortium of countries that collaborated in the development of Covax when it first started (41), but also did not anticipate the purchase of active pharmaceutical ingredients (AFI) produced by Chinese, North American, and European pharmaceutical companies. Further, nor did it organize its needles and syringes stocks (42). In addition, the current management of the Ministry of Health has not been structured to include a clear plan for vaccinating the Brazilian people against COVID-19 in the National Immunization Program (43).

It was only on 2020 December 16th that the Ministry of Health launched the national immunization plan against COVID-19 (44). In its first phase, this document includes, in addition to health professionals who work on the front lines of the fight against the pandemic, elderly people over 60 years old living in nursing homes, Indigenous Peoples over 18 years (45), as a result of the Indigenous organizations' movement. Despite the plan was launched in December, the first dose of the vaccine was offered on January 17th, in São Paulo state, 1 month later.

Regardless of the unprecedented health crisis that we are living in; the high rates of incidence, mortality, and lethality, reported here as well as high prevalence rates for Sars-CoV-2 antibodies for Indigenous Peoples (46); the cry of national and international society; and the pressure from Indigenous organizations and associations, Bolsonaro and his advisors continue to deny access to the best treatments available in the unified health system (SUS) to Indigenous Peoples living in urban areas or resumption areas and territories not regulated by FUNAI (2). The national immunization plan restricts the vaccine doses to approximately half of the Indigenous population, even though the action promoted by APIB in the Brazilian Supreme Court underpins that all Indigenous lives matter. In one movement, the federal government reinforces the structural racism historically present in the country, violates fundamental rights guaranteed in the Brazilian Constitution and international treaties, denies access to essential health care, and puts people under a situation of extreme vulnerability of falling ill and dying by COVID-19 (47).

In accordance with Charlier and Varison (2), we believe that there are only two solutions to guarantee the survival of Indigenous Peoples in the COVID-19 pandemic. First, taking into consideration the Indigenous movement and their association's view in order to elaborate public health policies regarding local perspectives on diseases, their determinants, as well as treatments culturally feasible. Then, the respect to the right to self-determination recognized by the Federal Constitution and by the UN Declaration on the Rights of Indigenous Peoples (2007). Otherwise, we will keep watching these peoples suffering and dying on the margins of society.

Indigenous Peoples are both grieving and fighting at the same time. Their resilience is their strength. The loss of an entire immaterial world of ancestral knowledge is occurring at a time when this very knowledge is of the utmost importance to fight this disease and move beyond it. Unfortunately, it seems that Brazil, as a whole, as well as the Indigenous population, keep under threat by the syndemic promoted by the coronavirus and federal government acting together. For all those who lost their lives in this pandemic, you will not be silenced.

## Data Availability Statement

The original contributions presented in the study are included in the article/Supplementary Material, further inquiries can be directed to the corresponding author/s.

## Author Contributions

MF, RL, and PB were responsible for the organization of the study and its conception. VP, MN, and MC contributed with the context, database, results, and conclusion. AA, IC, CS, and MB performed the statistical and geospatial analysis. All authors contributed to the revision of the manuscript, having read and approved the submitted version and have been working on the Indigenous agenda for a long period, particularly VP, MN, and MC belong to COIAB, and VP and MN are Indigenous leaders.

## Conflict of Interest

The authors declare that the research was conducted in the absence of any commercial or financial relationships that could be construed as a potential conflict of interest.

## Footnotes

^1^Pardo is a color and race definition used by the Brazilian Institute of Geography and Statistics (IBGE), and it denotes a mixed-race origin (10).

^2^For further information: https://covid.saude.gov.br/.

^3^For further information about the application “Indigenous Covid-19 Alert”: https://coiab.org.br/covid.

^4^SESAI manages three healthcare unit types: (i) basic healthcare stations, (ii) the so-called Polos-Base (PB), and (iii) the Indigenous Health Centers (CASAI).

^5^For further information: http://cnes.datasus.gov.br/.

^6^RAISG is a consortium of civil society organizations from the Amazon countries that produces and disseminates statistical data and geospatial socio-environmental information.

^7^CAR is the acronym for *Cadastro Ambiental Rural*, the mandatory register for every rural property to be recognized by the federal government.

^8^To calculate deforestation, we considered the whole area for each Indigenous Land and excluded rivers, lakes, and other types of landscape features that did not match the naturally occurring vegetation.

^9^For further information: https://bit.ly/3rGWL1M.

^10^For further information: http://apib.info/2020/08/01/adpf-709-no-supremo-povos-indigenas-e-o-direito-de-existir/.

^11^For further information: https://bit.ly/3eOYDQ.
